# Takotsubo Syndrome (Broken-Heart Syndrome or Stress Cardiomyopathy) in an Epileptic Pregnant Woman: A Case Report

**DOI:** 10.7759/cureus.36308

**Published:** 2023-03-17

**Authors:** Deemah K Harb, Widad Abdelkareem, Komal Hazari, Juwairia Alali, Asma Fahad, Rawan Al-Mekhlafi, Abeir Ammar

**Affiliations:** 1 Internal Medicine, Dubai Health Authority, Latifa Women and Children Hospital, Dubai, ARE; 2 Cardiology, Dubai Academic Health Corporation, Dubai, ARE; 3 Obstetrics and Gynaecology, Dubai Academic Health Corporation, Dubai, ARE; 4 Obstetrics and Gynaecology, Dubai Health Authority, Latifa Women and Children Hospital, Dubai, ARE

**Keywords:** heart failure, stress cardiomyopathy, epilepsy, pregnancy, takotsubo syndrome

## Abstract

Stress cardiomyopathy (Takotsubo syndrome) is a rare and transient cardiac dysfunction that has been reported in pregnant women with multiple triggering conditions. In general, those cases recovered from the acute cardiac insult within a few weeks.

We report a 33-year-old 22 weeks pregnant woman, who presented with an episode of status epilepticus and subsequently developed acute heart failure. She had full recovery within three weeks and continued her pregnancy till term. She became pregnant again two years after this initial insult, remained asymptomatic with stable cardiac function and had normal vaginal delivery at term.

## Introduction

Takotsubo syndrome was first reported in 1990 by the Japanese. The name is descriptive of the echocardiogram appearance of the left ventricle at the end of the systolic phase that appears like the Japanese octopus trap (tako for octopus, tsubo for pot) [1,2]. In a systematic review, the diagnosis of stress cardiomyopathy was up to 2% of the cases suspected with acute coronary syndrome or ST-elevation myocardial infarction [2]. There are few published case reports of Takotsubo cardiomyopathy during pregnancy [3,5]. Stress cardiomyopathy has been on the rise, but as the disease is underdiagnosed, the actual incidence in pregnancy remains unknown [3].

Various case reports of Takotsubo syndrome have been published following status epilepticus in the general population [6]. In a series of 50 reported cases, one-third were associated with seizures, out of which generalized seizures were the most common type [7].

The underlying pathology of Takotsubo syndrome is not fully understood. Various proposed mechanisms include excessive catecholamines, coronary artery spasms, and microvascular dysfunction. It was found to be associated more with psychiatric and neurological disorders [1,2].

Patients usually present with substernal chest pain, dyspnea, or syncope. In addition, they present with changes in the ST segment in the chest leads, significant rise of troponin levels, mild elevation of creatine kinase, and marked elevation of brain natriuretic peptide. Transient apical systolic left ventricular dysfunction is often found [1].

Supportive care and recovery from the physical or emotional insult often result in recovery of cardiac function; few patients develop complications such as shock and acute heart failure which may need ICU care [1,2].

## Case presentation

We report a case of a 33-year-old, gravida three, 22 weeks pregnant woman brought by ambulance to our emergency department in an unconscious state with an ongoing generalized tonic-clonic seizure for the last one hour before the presentation.

Upon arrival, she had a temperature of 37 °C, heart rate of 145 beats per minute, blood pressure of 113/76 mmHg, respiratory rate of 30 cycles per minute and arterial oxygen saturation (SaO2) of 100%. She received initial emergency management, including oxygen support, IV diazepam and magnesium sulfate.

She was intubated and transferred to the intensive care unit. She was administered IV levetiracetam 1000 mg followed by levetiracetam 2000 mg IV along with oxcarbazepine 900 mg twice daily through the Ryles tube. She was kept intubated and ventilated for the low threshold for recurrent seizures. 

The patient's past medical history revealed an established diagnosis of focal epilepsy (left frontotemporal) since the age of 21 years; she was compliant with medications and neuro-medical follow-up. She had remained seizure-free for nine months before the presentation. 

The bedside obstetric sonogram showed a single active fetus in cephalic presentation, with parameters corresponding to 22 weeks of gestation and an estimated fetal weight of 500 grams. There were no signs of placental abruption or subchorionic hematoma, and the amount of amniotic fluid was within the normal range.

**Figure 1:**
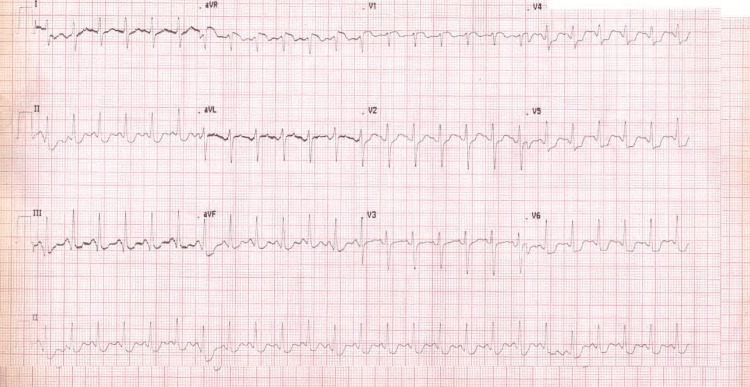
Post ICU transfer first ECG after hemodynamic deterioration showing sinus tachycardia with global non-specific ST-T changes.

Thirty minutes after arriving at the ICU, she had a sudden drop in blood pressure to 66/30 mm Hg, with heart rate reaching 150 beats/minute. She was managed with fluid challenge and required inotropes support with norepinephrine. Her ECG displayed sinus tachycardia with global non-specific ST-T changes (Figure 1), and her cardiac enzymes resulted in a significantly elevated troponin level.

**Figure 2:**
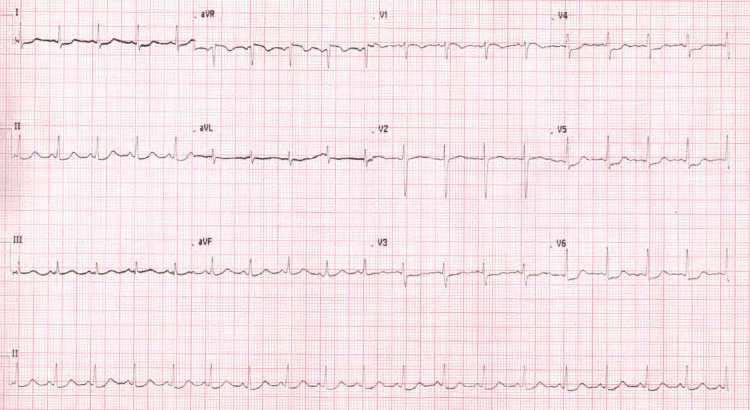
Repeat ECG 12 hours after ICU admission showing persistent global ST-T changes suggestive of cardiac injury.

**Figure 3:**
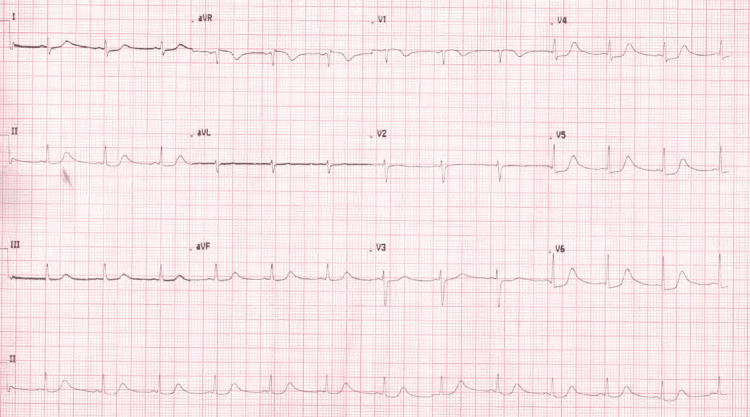
Repeat ECG day two post ICU admission showing moderate ST segment depression.

The monitoring of troponin and cardiac enzymes revealed a rising pattern (Table 1), and the serial ECGs were suggestive of acute cardiac injury (Figure 2, 3).

**Table 1:** Serial laboratory investigations during hospital stay CKMB: creatine kinase-MB, CPK: creatine phosphokinase, BNP: B-type natriuretic peptide

Lab Investigation	Reference Range	On admission	8 hours after admission	15 hours after admission	Day 2 of admission	Day 2 of admission	Day 3 of admission	Day 5 of admission
Troponin	<14ng/L	286	1260		942	545	394	
CPK	0-167 U/L	59				313		92
CKMB	0-24 U/L	23				44		
Pro BNP	<125pg/mL	24.3		1713	4146		5393	

Transthoracic echocardiography revealed dilated left ventricle (LV) and left atrium (LA), left ventricular ejection fraction (LVEF) 20-30%, moderate to severe mitral regurgitation (MR), Type 3 diastolic dysfunction, moderate tricuspid regurgitation (TR), and right ventricular systolic pressure (RVSP) 38 mm hg. Parenteral dobutamine, furosemide, and low molecular weight heparin were added to the management protocol.

CT scan brain was reported as normal; serial electroencephalograms (EEGs) showed bilateral slow waves, more on the right side, with some irritative discharges on the right frontocentral area.

The patient’s preliminary diagnosis was stress cardiomyopathy secondary to status epilepticus, and a multidisciplinary approach to management was carried out involving the concerned subspecialties of neurology, cardiology and obstetrics.

On the fifth day, inotropic medications were discontinued, and she was extubated. On the sixth day, she was transferred out of the ICU and continued on anti-failure medications including furosemide and bisoprolol. Angiotensin converting enzyme (ACE) inhibitors were not started as they are contraindicated in pregnancy.

On the ninth day, she was in well condition, asymptomatic, vitally stable and discharged home.

The follow-up echocardiogram at two and four weeks after discharge showed recovery of the left ventricular systolic function with an estimated EF of 50-55%, normal LV mass indexed with normal LV diastolic function, normal LA, thickened mitral leaflets with mild to moderate MR, trivial TR and normal PA systolic pressure 20 mmHg.

She had an uneventful normal vaginal delivery at 38 weeks with an infant weighing 2310 grams and an APGAR score of 8 and 9. She remained in a stable medical condition at three- and six-month follow-ups after delivery.

Two years later she presented again to our facility pregnant in her third trimester; she remained seizure-free and compliant to the antiepileptic medications at higher doses than the previous pregnancy, cardiac wise she remained asymptomatic with normal echocardiogram. She completed her pregnancy till term and had an uneventful normal vaginal delivery.

## Discussion

Our case is a 22 weeks pregnant epileptic patient who developed acute heart failure following an attack of status epilepticus. Our initial differential diagnosis included coronary artery disease, pulmonary embolism, and worsening preexisting cardiomyopathy or stress cardiomyopathy. Since our patient had completely recovered from the cardiac function within two weeks and remained well and asymptomatic, our final diagnosis was stress cardiomyopathy.

Generally, stress cardiomyopathy-induced heart failure is managed like heart failure due to other etiologies, including diuretics and beta blockers. ACE inhibitors or angiotensin receptor blockers (ARBs) were deferred for her due to pregnancy. The duration of anti-failure therapy is not yet well established; in our patient, we carried on for six weeks after the improvement of the cardiac function [8].

Takotsubo syndrome can occur at different intervals from the onset of acute insult [9]. In our case, it occurred within two to three hours, raising the possibility that pregnancy may have played a role in her profound response.

## Conclusions

Takotsubo syndrome is a rare condition during pregnancy, with most of the reported cases occurring during the late third trimester or in the postpartum period, but our patient had an early presentation at 22 weeks of gestation with acute stress cardiomyopathy-induced heart failure triggered by an attack of status epilepticus, she was intensively managed and carried on with her pregnancy till term; two years later she became pregnant, remained asymptomatic with uneventful antenatal and post-natal period.

Stress cardiomyopathy can have lethal sequelae, especially during the postictal period. Pregnant women following a seizure episode may not present the classic symptoms of acute cardiac injury; hence, seizure-induced stress cardiomyopathy can be missed or underdiagnosed. We recommend that the monitoring of the pregnant woman following a seizure episode may include close hemodynamics observation, serial ECG and cardiac enzymes with echocardiogram as indicated.
